# Clinical characteristics of virus-related uveitic secondary glaucoma: focus on cytomegalovirus and varicella zoster virus

**DOI:** 10.1186/s12886-022-02348-4

**Published:** 2022-03-22

**Authors:** Xintong Fan, Zhizhe Li, Ruyi Zhai, Qilian Sheng, Xiangmei Kong

**Affiliations:** 1 Eye Institute and Department of Ophthalmology, Eye & ENT Hospital, Fudan University, Shanghai, 200031 China; 2 NHC Key Laboratory of Myopia (Fudan University); Key Laboratory of Myopia, Chinese Academy of Medical Sciences, Shanghai, 200031 China; 3 Shanghai Key Laboratory of Visual Impairment and Restoration, Shanghai, 200031 China; 4 Department of Ophthalmology, Suzhou Municipal Hospital, The Affiliated Suzhou Hospital of Nanjing Medical University, Suzhou, Jiangsu China

**Keywords:** Uveitic glaucoma, Posner-Schlossman syndrome, Cytomegalovirus, Varicella zoster virus, Secondary glaucoma

## Abstract

**Background:**

We aimed to analyze the clinical characteristics of secondary glaucoma related to cytomegalovirus (CMV)- and varicella zoster virus (VZV)-positive uveitis.

**Methods:**

In this retrospective study, we enrolled patients with anterior uveitic secondary glaucoma. All the patients underwent aqueous and serum analyses for viral antibody through enzyme-linked immunosorbent assay. Among the 60 included patients, 22 had CMV-negative Posner-Schlossman syndrome (CMV-negative PSS), 25 had CMV-positive PSS, and 13 had VZV-positive anterior uveitis secondary glaucoma (VZV-AUSG). We evaluated the following main indicators: age, disease duration, intraocular pressure (IOP), cup-to-disc ratio, best corrected visual acuity (BCVA), corneal endothelial cell (CEC) count, ocular morphological changes, and medical treatments.

**Results:**

We found that 53.2% (25/47) patients with PSS were CMV-positive. Patients with CMV-positive PSS had a larger cup-to-disc ratio (*p* = .043), lower CEC density (*p* = .017), more severe CEC loss (*p* < .001), and more iris depigmentation (*p* = .006) than CMV-negative PSS patients. Compared with patients with CMV-positive PSS, those with VZV-AUSG were older (*p* = .003), presented a higher IOP (*p* = .015), and had poorer BCVA (*p* < .001). Patients with CMV-positive PSS and VZV-AUSG all accepted ganciclovir treatment, and those with CMV-positive PSS used fewer antiglaucoma agents simultaneously compared with CMV-negative PSS (*p* = .005) and VZV-AUSG (*p* < .001). All three groups had a comparable proportion of patients requiring antiglaucoma surgery.

**Conclusions:**

We observed some distinctive clinical features in CMV-positive PSS compared with CMV-negative PSS. Further, we found that patients with VZV-AUSG presented with a higher IOP and worse visual acuity, and required more antiglaucoma medication than those with CMV-positive PSS.

## Background

Posner-Schlossman syndrome (PSS), which is a secondary glaucoma subtype, was first described in 1948 and is characterized by unilateral, recurrent, acute attacks of mild non-granulomatous anterior uveitis accompanied by pronouncedly elevated intraocular pressure (IOP) [1]. With increasing recognition, PSS, which was initially considered benign, is now identified as a potential cause of chronic secondary glaucoma [2–4]. Cytomegalovirus (CMV), which belongs to the *Herpesviridae* family, has recently been established as a causative agent of anterior uveitis (AU) and corneal endothelitis [5–7]. Since CMV was first detected in the aqueous humor of patients with PSS in 1987 [8], there is strong evidence of a high prevalence of ocular CMV infection in patients with PSS [9–12].

However, there have been few studies that have elaborately compared patients with CMV-positive PSS and those with CMV-negative PSS. Most related studies have only performed a brief and tentative exploration of the possible role of CMV in PSS [13, 14]. It is crucial that clinicians are able to distinguish patients with CMV-positive PSS from those with CMV-negative PSS since corticosteroid treatment without antiviral treatment might cause exacerbation of CMV-positive PSS [6]. The diagnosis of CMV infection requires diagnostic testing of aqueous humor aspirates with an antibody assay or polymerase chain reaction (PCR) analysis. Both approaches are expensive and inaccessible to all patients, especially when patients decline an aqueous tap. Therefore, we aimed to thoroughly compare eyes with CMV-positive and CMV-negative PSS to improve their distinction and allow us to better understand this ocular disease.

Besides CMV, varicella zoster virus (VZV) and herpes simplex virus (HSV), which also belong to the *Herpesviridae* family, are also regarded as leading causes of infectious AU [15, 16]. We only found three patients with AU infected by HSV at the glaucoma clinic of our hospital. Therefore, we focused on patients with VZV-AUSG in our study due to the relatively rare infection of HSV we observed clinically. Previous studies have reported that it is relatively hard to diagnose existing herpetic uveitis in patients with VZV iritis without cutaneous eruption [17, 18]. In 13 patients with VZV-positive AU secondary glaucoma (VZV-AUSG) included in this study, only three cases had skin eruption, which can even account for more misdiagnosis. Further, AUSG has been reported to be often misdiagnosed as PSS [19]. Therefore, we deemed it necessary to conduct this study.

By analyzing the CMV-positive PSS characteristics, as well as the similarities and differences between CMV-positive PSS and VZV-related secondary glaucoma, we hope to help clinicians make an early diagnosis, as well as to allow a better understanding of these uveitis-relevant diseases.

## Methods

### Ethics statement

This retrospective study was approved by the Institutional Review Board of the Eye and ENT Hospital of Fudan University, Shanghai, China (no. 2017006–2), in accordance with the norms of the 1964 Declaration of Helsinki.

### Patients

**Fig. 1:**
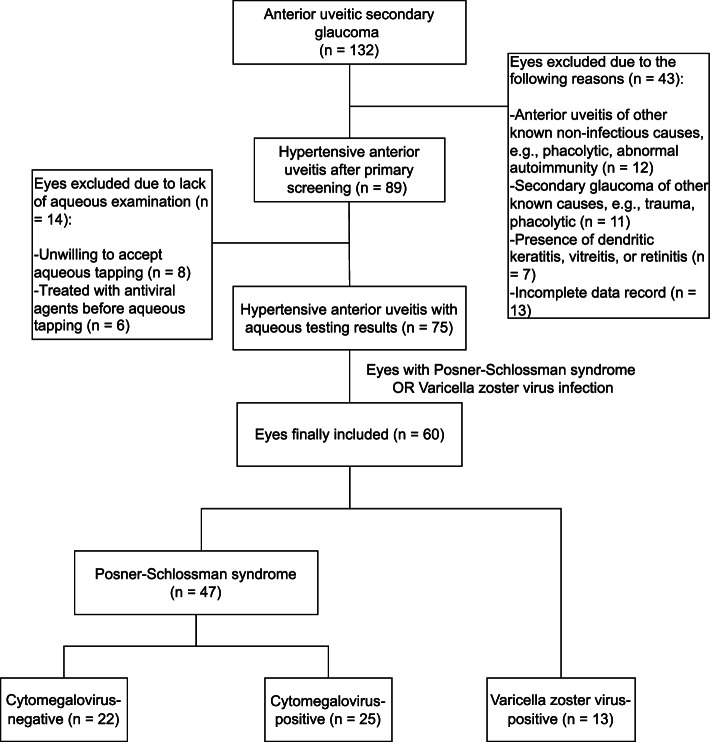
The flow chart of the patient inclusion

We enrolled patients who visited the glaucoma clinic at the Eye and ENT Hospital of Fudan University between January 2016 and August 2019. We used our electronic health records system and searched the diagnosis field of patients. The search terms we used were “anterior uveitic secondary glaucoma”, “anterior uveitis”, and “secondary glaucoma”. Patients diagnosed with “anterior uveitic secondary glaucoma” or diagnosed with both “anterior uveitis” and “secondary glaucoma” were identified. Finally, a total of 132 patients diagnosed with anterior uveitic secondary glaucoma were screened in the electronic records system of our hospital. The detailed flow chart of the patients’ inclusion in the study is shown in Fig. 1. Finally, a total of 60 patients were included in this study, including 47 patients with PSS and 13 VZV-AUSG. Among the enrolled patients with PSS, 25 were CMV-positive and 22 CMV-negative cases (also negative for herpes simplex virus and VZV). Three of CMV-positive and two of CMV-negative patients were bilateral. For bilateral cases, the more severe eye was included in this study. All the 13 patients with VZV-AUSG were unilateral. All the included patients were immunocompetent without human immunodeficiency virus (HIV) infection and they all had evidence or history of one episode. All the patients had previously received topical corticosteroid and antiglaucoma treatment; however, none had received antiviral therapy before the first visit to our glaucoma clinic where the aqueous humor was sampled. The exclusion criteria were as follows: 1) secondary glaucoma of other known causes; 2) anterior uveitis of other known non-infective causes, including abnormal autoimmunity and phacolytic factor; 3) presence of dendritic keratitis, vitreitis, or retinitis; 4) eyes treated with antiviral agents before aqueous tap; 5) incomplete data record. Topical 2% ganciclovir solution were applied to patients with CMV-positive PSS and VZV-AUSG 4 times per day during the follow-up period after receiving the results of aqueous viral antibody. The frequency of corticosteroid use was in accordance to the severity of ocular inflammation.

### Data collected

We reviewed the following demographic data of the patients from the medical records: gender, age at onset (first uveitis episode), age at diagnosis (CMV- or VZV-positive), laterality, disease duration (prior to aqueous tap), and attack frequency. The patients underwent thorough ophthalmologic examination based on their clinical requirements, including slit-lamp microscopy, indirect ophthalmoscopy, best corrected visual acuity (BCVA), IOP, visual field, and corneal endothelial cell count. Further, we collected the following information: cup-to-disc ratio, keratic precipitates (KPs), anterior chamber inflammation, clinical appearance of the iris, presence of cataracts, follow-up time, medical treatment at the first and last visit (including the use of antiglaucoma agents and corticosteroids), corticosteroid dependency, and surgical intervention. In patients with VZV-positive AUSG, we also obtained data regarding facial and periocular herpes zoster, conjunctival congestion, corneal edema, and pupillary configuration. Clinical data were noted at the time of the first visit of PSS and VZV-AUSG patients and after a short-time follow-up of PSS patients.

We measured the corneal endothelial cell (CEC) density in both diseased and healthy eyes and calculated the relative CEC loss rate as follows: CECs loss (%) = (1-CEC density in the diseased eye/CEC density in the healthy eye) × 100%. Iris depigmentation was determined when iris color of the disease eye was lighter than the fellow eye, which was observed by slit-lamp microscopy.

### Diagnosis of PSS and AUSG

The diagnostic criteria of PSS were based on clinical manifestations, including recurrent anterior chamber inflammation, elevated IOP during the attack, diffuse cornea edema and characteristic KPs, and the absence of anterior or posterior synechiae. Between attacks, the IOP is usually normal and the anterior chamber angles are constantly open.

The diagnosis criteria of VZV-AU were based on a positive aqueous virus antibody test and clinical manifestations, including keratitis, elevated IOP, iris sector atrophy developing over time, and facial or periocular varicella zoster with subsequent keratouveitis. Regarding patients with AU, secondary glaucoma was defined as elevated IOP (> 21 mmHg) with optic disc abnormalities. The diagnosis of secondary glaucoma was confirmed by a glaucoma specialist (XK).

### Test of aqueous viral antibody

After aqueous sample collection, we used enzyme-linked immunosorbent assay (ELISA, Virion/Serion, Germany) to detect CMV and VZV IgG antibodies. Further, we corrected the viral antibody in the aqueous humor using aqueous and serum albumin as follows: (aqueous CMV or VZV IgG/serum CMV or VZV IgG)/ (aqueous albumin concentration/serum albumin concentration). The corrected ratio was abbreviated as s/co. The result was considered positive when viral IgG was detected in aqueous humor and the aqueous humor/serum correction ratio was larger than 0.6 s/co. The diagnostic performance of the corrected ratio on ocular virus infection has been evaluated by the ROC curve [20–22]. The area under the ROC curve for the corrected ratio was 0.942 (95% *CI*: 0.859 to 0.984) as described in our previous study [22].

### Statistical analysis

Data were expressed as mean with 95% C.I. We used the χ^2^ test or Fisher’s exact test to compare proportions, when appropriate. We used the Mann-Whitney U test for between-group comparisons of continuous variables. We used SPSS ver. 26 to perform statistical analysis and between-group differences were considered significant at *P* < .05.

## Results

### Ocular clinical features of CMV-positive and CMV-negative Posner-Schlossman syndrome

Table 1 presents the demographic data and basic characteristics of both patients with CMV-positive and CMV-negative PSS. There was no significant between-group difference in the basic characteristics, including the age at the first onset, age at the clinic visit, gender, eye literality, and disease duration (*p* = .975, *p* = .654, *p* = 730, *p* = .654, and *p* = .115, respectively).

**Table 1:** Demographic data and basic characteristics of patients with Posner-Schlossman syndrome

Characteristics	CMV-Negative Patients (***N*** = 22)	CMV-Positive Patients (***N*** = 25)	Total Group (***N*** = 47)	***P*** Value^**a**^
Age at the first onset (years)	32.5 (27.1–37.9)	31.8 (27.0–36.6)	32.1 (28.7–35.6)	.975
Age at the clinic visit (years)	36.9 (31.5–42.3)	39.0 (33.4–44.6)	38.0 (34.2–41.8)	.654
Male gender	13 (59.1)	16 (64.0)	29 (61.7)	.730
Bilateral	2 (9.1)	3 (12.0)	5 (10.6)	1.000
Eye involvement				.654
Right	12 (54.5)	12 (48.0)	24 (51.1)	
Left	10 (45.5)	13 (52.0)	23 (48.9)	
Duration of disease (years)	4.4 (2.4–6.4)	7.2 (4.5–9.9)	5.9 (4.2–7.6)	.115
< 12 months	6 (27.3)	4 (16.0)	10 (21.3)	
12–120 months	12 (54.5)	16 (64.0)	28 (59.6)	
> 120 months	4 (18.2)	5 (20.0)	9 (19.2)	

Data are expressed as mean (95% C.I) or number of patients (%)

^a^χ^2^ test or Fisher’s exact test was used to compare proportions between CMV-negative and CMV-positive PSS; Mann-Whitney U test was used to compare continuous variables between CMV-negative and CMV-positive PSS

Table 2 presents the ocular manifestations of the patients with CMV-positive and CMV-negative PSS. Compared with patients with CMV-negative PSS, patients with CMV-positive PSS exhibited a significantly larger cup-to-disc ratio and higher CECs loss rate (*p* = .043 and *p* = .001, respectively). Regarding ocular morphological alterations, 20/25 (80%) and 9/22 (40.9%) of the patients in the CMV-positive and CMV-negative PSS groups, respectively, had iris depigmentation, which was a significant proportion (*p* = .006). There was no significant between-group difference in the IOP and BCVA (*p* = .839 and *p* = .554, respectively). Further, the proportion of eyes with mutton-fat KPs, coin-shaped KPs, Tyndall effect, and cataract was also similar between these two groups (*p* = .095, *p* = .894, *p* = 1.000, and *p* = .085, respectively).

**Table 2:** Comparison of ocular clinical features of patients with CMV-positive and CMV-negative Posner-Schlossman syndrome

Characteristics	CMV-Negative Patients (***N*** = 22)	CMV-Positive Patients (***N*** = 25)	***P*** Value^**a**^
IOP at the clinic visit (mm Hg)	29.6 (24.5–34.8)	29.0 (25.0–33.0)	.839
Peak IOP (mm Hg)	48.5 (44.4–52.6)	49.2 (41.3–57.1)	.235
Cup-to-disc ratio	0.5 (0.4–0.5)	0.6 (0.5–0.6)	.043*
Corrected visual acuity			
logMAR	0.1 (0.02–0.2)	0.3 (0.01–0.6)	.554
Decimal (minutes)	0.8 (0.7–0.9)	0.8 (0.6–0.9)	.554
CEC density of the diseased eye (cell/mm^2^)	2490.6 (2320.8–2660.5)	2196.3 (2020.9–2371.8)	.017*
CEC destiny of the healthy eye (cell/mm^2^)	2756.6 (2596.3–2916.9)	2828.5 (2702.2–2954.8)	.448
Relative CEC loss (%)	9.6 (5.9–13.3)	22.1 (16.3–27.9)	.001*
KPs			
-Mutton-fat	19 (86.36)	25 (100)	.095
-Coin-shaped	5 (22.7)	6 (24.0)	.894
Tyndall effect	3 (13.6)	3 (12.0)	1.000
Iris depigmentation	9 (40.9)	20 (80.0)	.006*
Cataract	6 (27.3)	13 (52.0)	.085

Data are expressed as mean (95% C.I) or number of patients (%)

*IOP* Intraocular pressure, *CEC* Corneal endothelial cell, *KPs* Keratic precipitates

^a^χ^2^ test or Fisher’s exact test was used to compare proportions between CMV-negative and CMV-positive PSS; Mann-Whitney U test was used to compare continuous variables between CMV-negative and CMV-positive PSS

*p < .05

### Medical treatment and follow-up outcomes of the patients with CMV-positive and CMV-negative PSS

Table 3 presents the applied treatments upon diagnosis with CMV-positive PSS and the relevant outcomes after a follow-up period. The CMV-positive group used fewer IOP-lowering agents at the initial visit compared with the CMV-negative group (*p* = .005). All patients with CMV-positive PSS were treated with topical 2% ganciclovir during the follow-up. After an approximately equal follow-up duration (mean: 4.9 and 5.9 weeks, respectively), the IOP of all patients in both groups, except two who were CMV-negative, was within the normal range after medical treatment (< 21 mmHg). Further, the IOP of patients with CMV-positive PSS was lower than that of CMV-negative PSS (*p* = .016), indicating that fewer antiglaucoma agents were sufficient to control the IOP in patients in the CMV-positive group with the treatment of topical 2% ganciclovir. Further, there was no significant between-group difference in the mutton-fat KPs, coin-shaped KPs, and Tyndall effect after the follow-up. Moreover, at the end of the short-term follow-up, there was complete disappearance of the KPs of 10 (52.6%) CMV-positive and 10 (40%) CMV-negative eyes, respectively. Tyndall effect turned negative in all the patients in both groups.

**Table 3:** Medical treatment and outcome of treatment between patients with CMV-positive and CMV-negative Posner-Schlossman syndrome

Characteristics	CMV-Negative Patients (***N*** = 22)	CMV-Positive Patients (***N*** = 25)	***P*** Value^**a**^
Medical treatment at the first visit			
Number of patients treated with antiglaucoma medication	20 (90.9)	15 (60)	.015*
Number of antiglaucoma agents used simultaneously	2.0 (1.5–2.4)	1.0 (0.6–1.5)	.005*
Frequency of corticosteroid eye drop (times per day)	2.5 (2.1–2.9)	2.8 (2.5–3.1)	.271
Number of patients with corticosteroid dependency	13 (59.1)	12 (48)	.447
Ocular outcomes at the end of follow-up			
IOP (mm Hg)	16.7 (15.0–18.3)	14.4 (13.2–15.7)	.016*
IOP reduction (mm Hg) (compared with the IOP at the first clinic visit)	13.0 (7.8–18.1)	14.6 (10.4–18.8)	.129
mutton-fat KPs	9 (40.9)	15 (60)	.191
Coin-shaped KPs	1 (4.5)	0 (0)	.281
Tyndall effect	0 (0)	0 (0)	NA
Medical treatment at the end of follow-up			
Number of patients treated with antiglaucoma medication	11 (50)	8 (32)	.210
Number of antiglaucoma agents used simultaneously	1.0 (0.5–1.5)	0.5 (0.2–0.9)	.148
Frequency of corticosteroid eye drop (times per day)	0.5 (0.2–0.9)	1.0 (0.5–1.4)	.213
Duration of follow-up (weeks)	4.9 (3.8–6.0)	5.9 (4.7–7.2)	.202

Data are expressed as mean (95% C.I) or number of patients (%)

*IOP* intraocular pressure, *KPs* keratic precipitates, *NA* not applicable

^a^χ^2^ test or Fisher’s exact test was used to compare proportions between CMV-negative and CMV-positive PSS; Mann-Whitney U test was used to compare continuous variables between CMV-negative and CMV-positive PSS

*p < .05

### Surgical intervention of the patients with CMV-positive and CMV-negative PSS

After 1 year follow-up, 8 eyes (32%) of CMV-positive PSS underwent antiglaucoma surgery eventually, including trabeculectomy in 3 eyes, aqueous drainage device implantation in 3 eyes, combined drainage device with phacoemulsification & intraocular lens implantation (CDPI) in 2 eyes. 2 eyes (9.09%) of CMV-negative PSS underwent trabeculectomy and CDPI, respectively. There was no statistically significant difference between the two groups (*p* = .079).

### Comparison of clinical features of patients with VZV-positive and CMV-positive secondary glaucoma

Further, we analyzed 13 patients with VZV-positive AU with secondary glaucoma. Table 4 presents their basic characteristics.

**Table 4:** Characteristics of patients with secondary glaucoma induced by VZV-positive anterior uveitis

Characteristics	VZV-Positive Patients (***N*** = 13)
Age (years)	55.1 (45.0–65.2)
Male gender	8 (61.5)
Duration of disease (months)	3.0 (0.3–5.6)
Eye involvement	
Unilateral	13 (100)
Bilateral	0 (0)
Facial herpes zoster	3 (23.1)
Periocular herpes zoster	3 (23.1)
Conjunctival congestion	10 (76.9)
Corneal edema	6 (46.2)
KPs	13 (100)
Iris depigmentation	13 (100)
Abnormal pupillary configuration	13 (100)

Data are expressed as mean (95% C.I) or number of patients (%)

*KPs* keratic precipitates

**Fig. 2:**
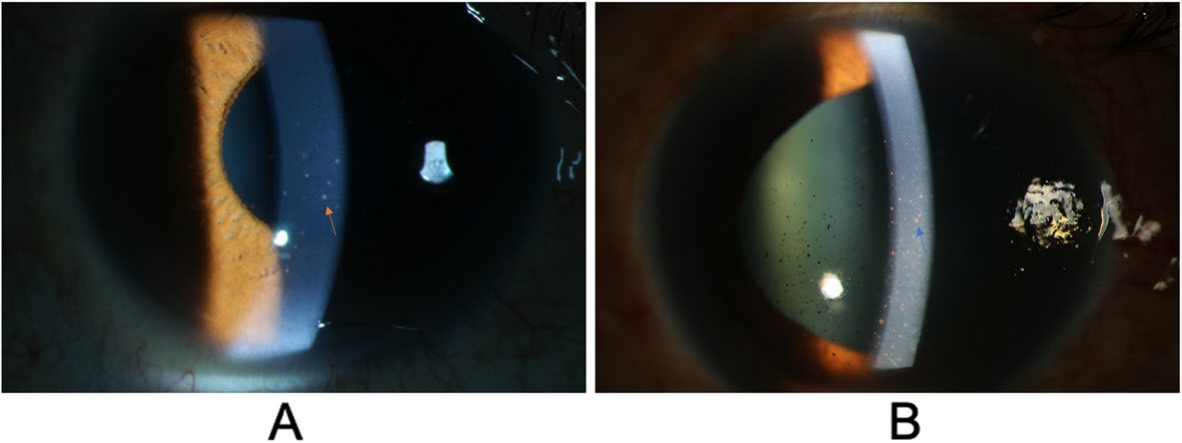
Typical morphology of keratic precipitates (KPs) in patients with cytomegalovirus (CMV)- and varicella zoster virus (VZV)-positive secondary glaucoma. **A** The yellow arrow indicates mutton-fat KPs observed in CMV-positive patients. **B** The blue arrow indicates pigmented KPs observed in VZV-positive patients

Table 5 presents the similarities and differences between patients with VZV-AUSG and those with CMV-positive PSS. The patients with VZV-AUSG were significantly older than those with CMV-positive PSS (*p* = .003). The patients with VZV-AUSG manifested significantly higher IOP (*p* = .015) and worse BCVA compared to those with CMV-positive PSS (*p* < .001). The visual acuity of 2 of the 13 patients in the VZV-AUSG group was finger count, which corresponded to LogMAR visual acuity 2.0. The visual acuity of 1 of the 25 patients in the CMV-positive PSS group was hand movement, which corresponded to LogMAR visual acuity 3.0. Mutton-fat KPs were more often seen in patients with CMV-positive PSS, while characteristic pigmented KPs were more often seen in patients with VZV-AUSG (shown in Fig. 2). Additionally, the prevalence of Tyndall effect was higher in the VZV-AUSG group (*p* < .001). Consistent with their higher IOP, patients with VZV-AUSG were treated with more antiglaucoma agents simultaneously than those with CMV-positive PSS (*p* < .001); however, there was no significant between-group difference in the application of corticosteroid. 4 of 13 eyes (30.8%) with VZV-AUSG received antiglaucoma surgery, including trabeculectomy in 3 eyes and CDPI in 1 eye. The surgical treatment of CMV-positive PSS has been detailed described before. The proportion of eyes requiring antiglaucoma surgery was similar between CMV-positive PSS and VZV-AUSG.

**Table 5:** Comparison of clinical characteristics of patients with VZV-positive and CMV-positive secondary glaucoma

Characteristics	VZV-Positive Patients (***N*** = 13)	CMV-Positive Patients (***N*** = 25)	***P*** Value^**a**^
Age (years)	55.1 (45.0–65.2)	39.0 (33.4–44.6)	.003*
Male gender	8 (61.5)	16 (64)	1.000
Laterality (right)	9 (69.2)	12 (48)	.307
IOP (mm Hg)	32.2 (27.2–37.3)	29.0 (25.0–33.0)	.015*
Cup-to-disc ratio	0.4 (0.3–0.6)	0.6 (0.5–0.6)	.093
Corrected visual acuity			
logMAR	0.8 (0.4–1.3)	0.3 (0.01–0.6)	.000*
Decimal (minutes)	0.3 (0.1–0.4)	0.8 (0.6–0.9)	.000*
KPs	13 (100)	25 (100)	NA
Tyndall effect	10 (76.9)	3 (12)	.000*
Iris depigmentation	13 (100)	20 (80)	.144
Cataract	9 (69.2)	13 (52)	.490
Medical treatment at the first visit			
Number of patients treated with antiglaucoma medication	12 (92.3)	15 (60)	.060
Number of antiglaucoma agents used simultaneously	2.4 (1.9–2.9)	1.0 (0.6–1.5)	.001*
Frequency of corticosteroid eye drop (times per day)	3.2 (2.7–3.6)	2.8 (2.5–3.1)	.110
Surgical intervention	4 (30.8)	10 (40)	.728

Data are expressed as mean (95% C.I) or number of patients (%)

*KPs* keratic precipitates, *IOP* intraocular pressure

^a^χ^2^ test or Fisher’s exact test was used to compare proportions between VZV-positive and CMV-positive secondary glaucoma; Mann-Whitney U test was used to compare continuous variables between VZV-positive and CMV-positive secondary glaucoma

*p < .05

## Discussion

In this study, we conducted detailed comparisons of patients with CMV-positive PSS and those with CMV-negative PSS; further, we also analyzed the different clinical features of VZV-positive and CMV-positive secondary glaucoma.

Among the enrolled patients with PSS, 53.2% were found to be CMV-positive. This proportion is similar to that reported by Chee et al. in 2008 [14]. However, inconsistent with our findings, Chee et al. did not report clinically detectable differences between the eyes of patients with CMV-negative and CMS-positive PSS. We found that patients with CMV-positive PSS had a larger cup-to-disc ratio and a more severe CECs loss rate, which is consistent with the findings of Su et al. in 2014 [23]. Moreover, the prevalence of iris depigmentation was higher in the CMV-positive group compared to that in the CMV-negative group. Chee et al. attributed their negative results to uncertainty of the PSS diagnosis and the underestimation of CMV infection among the eyes with PSS [14]. Moreover, we speculated that the indicators evaluated by Chee et al. might be inadequate to illustrate the clinical differences between CMV-positive and CMV-negative patients. Our findings are consistent with those of Su et al. where patients with CMV-positive PSS suffered more serious CEC loss than those with CMV-negative PSS [23]. In addition, Su et al. reported that patients with CMV-positive PSS presented worse BCVA than that in those with CMV-negative PSS. However, we did not find a significant between-group difference in the BCVA. This inconsistency is probably due to the fact that the average visual acuity of our patients was better than that of the patients enrolled by Su et al. Since our patients presented relatively minor visual impairment, we cannot rule out the possibility that the worsening of visual acuity with disease progression is worse in CMV-positive eyes than that in CMV-negative eyes.

Previous studies have reported a comparable IOP in patients with CMV-positive PSS and those with CMV-negative PSS, which is consistent with our findings [14, 23]. Further, patients in the CMV-positive group received fewer antiglaucoma agents at the first clinic visit than those in the CMV-negative group. After a short-term follow-up period, the IOP of patients with CMV-positive PSS was lower than that of CMV-negative PSS (*p* = .016), indicating that fewer antiglaucoma drugs were sufficient for controlling the IOP in CMV-positive patients with the treatment of topical 2% ganciclovir. This might be attributed to the more effortless IOP control in CMV-positive patients since they received etiological therapy; specifically, topical ganciclovir, targeting ocular viral infection.

In patients with PSS, surgical treatment is necessary when IOP becomes difficult to control with antiglaucoma medication alone. In all the 47 patients with PSS, 10 underwent antiglaucoma surgery; among them, 8 (80%) were CMV-positive. Su et al. observed a higher number of eyes requiring glaucoma surgery in patients with CMV-positive PSS [23]. In the current study, there was a tendency that more patients with CMV-positive PSS were treated antiglaucoma surgery, but the result did not reach statistical significance. Previous study indicated that CMV-positive eyes with longer disease duration (over 5 years) were more likely to receive glaucoma surgery compared with CMV-positive eyes with shorter duration of disease [23]. Therefore, the negative result may part of be attributable to the relative short disease duration of patients included in this study.

Among our enrolled patients with VZV-positive uveitis glaucoma, only 3 (23.1%) had facial herpes zoster, which is regarded as a strong indication of VZV-uveitis. It could be hard to correctly diagnose herpetic uveitis in patients with zoster sine herpete [17]. Further, Nakamura et al. reported a patient with bilateral iridocyclitis with elevated IOP unresponsive to steroid treatment who was finally diagnosed with zoster sine herpetic anterior uveitis with secondary glaucoma [24]. The iridocyclitis and secondary glaucoma were well controlled by acyclovir treatment and trabeculectomy [24]. Obviously, it is critical that patients with VZV-AU promptly receive medication after being diagnosed to avoid further complications.

Sungur et al. reported that secondary glaucoma was a frequent complication of viral uveitis, which has a good prognosis and rarely requires surgical intervention [25]. In the present study, all the patients with VZV-positive AU presented with secondary glaucoma. We assessed differences in the clinical manifestations between VZV-AUSG and CMV-positive PSS to allow better clinical distinction between the two diseases. A previous study reported that patients with CMV-AU were older than patients with VZV-AU patients [19]. In contrast, we found that patients with CMV-positive PSS were much younger than patients with VZV-AUSG. Moreover, we observed that anterior chamber inflammation in VZV-AUSG was more severe than that in CMV-positive PSS, which is consistent with previous findings that inflammatory severities in VZV-AU were higher than those in CMV-AU [19, 26]. Further, previous studies have reported a comparable decrease in vision in both patients with VZV-AU and CMV-AU [19, 26]. Contrastingly, we found that the visual acuity in VZV-AUSG was significantly poorer than that in CMV-positive PSS.

Our study has several limitations. First, we began observation at uveitis-related secondary glaucoma; given the relatively short follow-up time, we could not observe further complications that might have occurred with further progression. Second, the 95% CI for IOP showed that there is an overlap of values (upper and lower limits of IOP in both groups) for the two groups, which is probably due to the small sample size. Third, we did not perform PCR analysis to test CMV and VZV DNA in the aqueous humor, which might have led to an inaccurate rate of aqueous virus positivity. However, PCR assay does not confirm productive infection and could give a false positive result despite its high sensitivity [27]. Moreover, DNA examination tends to give a positive result at outset while antibody testing can give a positive result at any time point; however, both tests contribute to a precise diagnosis [27, 28]. A previous study indicated that combining PCR and Goldmann-Witmer coefficient (GWc) were contributive for the diagnosis of CMV anterior uveitis. And repeated tap may be suggested in case of very high clinical suspicion and negative results [29].

## Conclusions

In conclusion, compared with patients with CMV-negative PSS, those with CMV-positive PSS had a larger cup-to-disc ratio, more severe CECs loss, and greater iris depigmentation. Moreover, we identified distinctive features between VZV-AUSG and CMV-positive PSS. Early diagnosis and appropriate therapy could alleviate the risk of advanced glaucoma and eliminate the need for glaucoma surgery. Our findings might aid clinicians better understanding these entities.

## Data Availability

The datasets used and analysed during the current study are available from the corresponding author on reasonable request.

## Abbreviations

PSS: Posner-Schlossman syndrome
IOP: Intraocular pressure
CMV: Cytomegalovirus
AU: Anterior uveitis
PCR: Polymerase chain reaction
VZV: Varicella zoster virus
VZV-AUSG: VZV-positive AU secondary glaucoma
BCVA: Best corrected visual acuity
KPs: Keratic precipitates
CEC: Corneal endothelial cell
CDPI: Combined drainage device with phacoemulsification & intraocular lens implantation
